# A Risk Score Model Incorporating Three m6A RNA Methylation Regulators and a Related Network of miRNAs-m6A Regulators-m6A Target Genes to Predict the Prognosis of Patients With Ovarian Cancer

**DOI:** 10.3389/fcell.2021.703969

**Published:** 2021-09-23

**Authors:** Qian Li, Chen-Chen Ren, Yan-Nan Chen, Li Yang, Feng Zhang, Bao-Jin Wang, Yuan-Hang Zhu, Fei-Yan Li, Jun Yang, Zhen-An Zhang

**Affiliations:** ^1^Department of Gynecology, The Third Affiliated Hospital of Zhengzhou University, Zhengzhou, China; ^2^Henan Province Women and Children’s Hospital, Zhengzhou, China; ^3^The Third Affiliated Hospital of Zhengzhou University, Zhengzhou, China; ^4^Henan International Joint Laboratory of Ovarian Malignant Tumor, Zhengzhou, China

**Keywords:** ovarian cancer, m6A, RNA methylation, risk model, prognosis

## Abstract

Ovarian cancer (OC) is the leading cause of cancer-related death among all gynecological tumors. N6-methyladenosine (m6A)-related regulators play essential roles in various tumors, including OC. However, the expression of m6A RNA methylation regulators and the related regulatory network in OC and their correlations with prognosis remain largely unknown. In the current study, we obtained the genome datasets of OC from GDC and GTEx database and analyzed the mRNA levels of 21 key m6A regulators in OC and normal human ovarian tissues. The expression levels of 7 m6A regulators were lower in both the OC tissues and the high-stage group. Notably, the 5-year survival rate of patients with OC presenting low VIRMA expression or high HNRNPA2B1 expression was higher than that of the controls. Next, a risk score model based on the three selected m6A regulators (VIRMA, IGF2BP1, and HNRNPA2B1) was built by performing a LASSO regression analysis, and the moderate accuracy of the risk score model to predict the prognosis of patients with OC was examined by performing ROC curve, nomogram, and univariate and multivariate Cox regression analyses. In addition, a regulatory network of miRNAs-m6A regulators-m6A target genes, including 2 miRNAs, 3 m6A regulators, and 47 mRNAs, was constructed, and one of the pathways, namely, miR-196b-5p-IGF2BP1-PTEN, was initially validated based on bioinformatic analysis and assay verification. These results demonstrated that the risk score model composed of three m6A RNA methylation regulators and the related network of miRNAs-m6A regulators-m6A target genes is valuable for predicting the prognosis of patients with OC, and these molecules may serve as potential biomarkers or therapeutic targets in the future.

## Introduction

According to the Global Burden of Disease Study in 2018, ovarian cancer (OC) is the leading cause of cancer-related deaths among all gynecological tumors (Torre et al., 2018). Because of its rare early symptoms and lack of biomarkers for early clinical detection, more than 70% of patients with OC are first diagnosed at an advanced stage and have a relatively low 5-year overall survival rate of 30% (Colombo et al., 2006; Karnezis et al., 2017). During the past few decades, advanced therapeutic modalities have not significantly changed the survival rate of patients with OC, and relapse and chemoresistance are still the two major obstacles in OC treatment (Kim et al., 2018; Luvero et al., 2019). Therefore, an urgent need is to identify novel biomarkers and therapeutic targets for the early diagnosis and prognostic prediction of OC (Gupta et al., 2019).

Epigenetic modification is an extensive and important way of regulating cell physiological and pathological processes (Nebbioso et al., 2018). In recent years, RNA modifications, including *N6*-methyladenosine (m6A), *N1*-methyladenosine (m1A) and 5-methylcytosine (m5C), have been identified to play vital roles in the regulation of gene expression and function, similar to the modification of DNA and proteins (Roundtree et al., 2017). Of these modifications, m6A is a common posttranscriptional modification of RNA that is considered the most abundant internal chemical modification of mRNAs. Similar to DNA modification, m6A RNA methylation is dynamically regulated by the corresponding m6A regulators, which are well characterized into three subtypes, namely, “methylases-writers (including METTL3, METTL14, and WTAP),” “demethylases-erasers (including FTO and ALKBH5),” or “m6A binding proteins-readers (YTHDF1, eIF3, and IGF2BP1),” depending on different functions (Yang et al., 2018; Chen et al., 2019; Barbieri, 2020). Notably, m6A has been extensively reported to be involved in a variety of critical steps in RNA metabolism, such as RNA stability, mRNA splicing, translation, and degradation (Wang et al., 2014), and further influences cell processes and physiological functions, as well as the initiation and progression of cancers (Dai et al., 2018), including gastric cancer (Zhang et al., 2020), lung cancer (Xu et al., 2021), hepatic carcinoma (Lu et al., 2020), and breast cancer (Wang Y. et al., 2020). Some reports of m6A RNA methylation in OC have been published. For example, Ma Z et al. found that high expression of the “writer” METTL3 indicated increased malignancy and shorter survival of patients with EEOC by modulating aberrant m6A RNA methylation, which may be a potential therapeutic target in EEOC (Ma et al., 2020). Huang et al. (2020b) reported that “eraser” FTO-dependent m6A modifications inhibit OC stem cell self-renewal by blocking cAMP signaling. According to Li et al. (2020), the “reader” YTHDF2, a protein whose expression is repressed by miR-145, regulates proliferation, apoptosis, and migration in OC cells. However, the gene signatures and prognostic values associated with m6A regulators in the regulation of OC development and progression remain largely unexplored.

In the current study, we comprehensively analyzed the expression, clinicopathological characteristics, and prognostic relevance of 21 widely reported key m6A RNA methylation regulators based on an extensive bioinformatic analysis of the public genome databases GDC and GTEx. The expression of the m6A regulators VIRMA and HNRNPA2B1 predicted the prognosis of patients with OC. Moreover, we built a risk score model based on three selected m6A regulators (VIRMA, IGF2BP1, and HNRNPA2B1) using a LASSO Cox regression analysis and examined the moderate accuracy of the risk score model by performing ROC curve, nomogram, and univariate and multivariate Cox regression analyses. In addition, we constructed a regulatory network of miRNAs (predicted from miRBase)-m6A regulators-m6A target genes (predicted from m6Avar), including two miRNAs, three m6A regulators, and forty-seven mRNAs, and initially validated one of the pathways, namely, miR-196b-5p-IGF2BP1-PTEN, in OC based on the bioinformatic analysis and assay verification, which might represent a potential biomarker or therapeutic target in OC in the future.

## Materials and Methods

### Datasets

**FIGURE 1:**
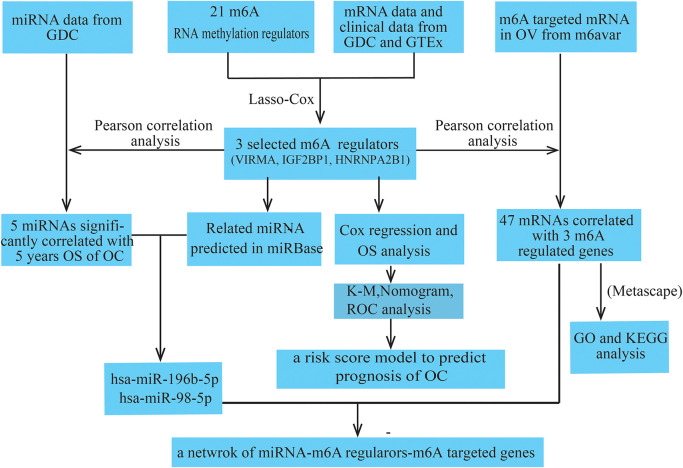
Workflow and analysis strategy used in the current study.

All datasets used in this study were publicly available, and the workflow of the current study is shown in Figure 1. RNA-seq transcriptome data (FPKM), including mRNA and miRNA expression data, and the corresponding clinicopathological information of 374 patients with OC were obtained from the GDC database^1^, and the RNA-seq transcriptome data of 88 normal human ovarian tissues were obtained from the GTEx database^2^. Detailed clinical information for the approximately 374 OC samples is shown in Table 1.

**TABLE 1:** The clinical information of 374 ovarian patients in GDC dataset.

Characteristics	OV patients (*N* = 374)	NO.%
**Age**		
<65	243	64.97%
≥65	129	34.49%
Unknow	2	0.53%
Stage		
Stage I	1	0.27%
Stage II	22	0.59%
Stage III	290	77.54%
Stage IV	57	15.24%
Unknow	4	1.07%
**Grade**		
G1	1	0.27%
G2	41	10.96%
G3	319	85.29%
G4	1	0.27%
GB	2	0.53%
GX	6	1.60%
Unknow	4	1.07%
**Tumor residual disease**		
No Macroscopic disease	66	17.65%
1–10 mm	170	45.45%
11–20 mm	25	6.68%
>20 mm	69	18.45%
Unknow	44	11.76%
**Vital status**		
Alive	171	45.72%
Dead	200	53.48%
Unknow	3	0.80%

### Selection and Analysis of the Differential Expression of m6A Regulators

We collected a list of 21 key m6A RNA methylation regulators from recently published studies (Zaccara et al., 2019), including 11 readers, 7 writers, and 3 erasers (Table 2). Then, we systematically compared the expression of these m6A regulators in ovarian tissues with different clinicopathological characteristics. All data were processed using R software (version 3.4.0). The Wilcoxon test was used to identify differentially expressed genes (DEGs) between the OC samples and non-cancerous samples.

**TABLE 2:** The description of 21 m6A RNA methylation regulators from publications.

Gene	Ensembl	Type	Gene	Ensembl	Type
METTL3	ENSG00000165819	Writers	YTHDF2	ENSG00000198492	Readers
METTL14	ENSG00000145388	Writers	YTHDF3	ENSG00000185728	Readers
METTL16	ENSG00000127804	Writers	IGF2BP1	ENSG00000159217	Readers
ZC3H13	ENSG00000123200	Writers	IGF2BP2	ENSG00000073792	Readers
WTAP	ENSG00000146457	Writers	IGF2BP3	ENSG00000136231	Readers
RBM15	ENSG00000162775	Writers	HNRNPC	ENSG00000092199	Readers
VIRMA	ENSG00000164944	Writers	HNRNPA2B1	ENSG00000122566	Readers
RBMX	ENSG00000147274	Readers	FTO	ENSG00000140718	Erasers
YTHDC1	ENSG00000083896	Readers	ALKBH3	ENSG00000166199	Erasers
YTHDC2	ENSG00000047188	Readers	ALKBH5	ENSG00000091542	Erasers
YTHDF1	ENSG00000149658	Readers			

### Construction of a Risk Score Model to Predict the Prognosis of Patients With OC

Using the glmnet function in the glmnet package to perform the LASSO Cox regression analysis, three genes serving as variables (VIRMA, IGF2BP1, and HNRNPA2B1) were selected to build the prognostic prediction model and visualized using the ggrisk package. The high-risk subtype (samples with a risk score greater than –0.03) and the low-risk subtype (samples with a risk score less than –0.03) were defined in OC cases based on the risk score of tumor samples in the train dataset. Also the high-risk subtype (samples with a risk score greater than –0.05) and the low-risk subtype (samples with a risk score less than –0.05) were confirmed in OC cases based on the risk score of tumor samples in the test dataset. Survminer and survival packages were further used to construct Kaplan–Meier (KM) survival curves. The effects of the three selected genes and constructed survival models on the 5-year survival rate of patients with OC were analyzed and evaluated using the log-rank test. Moreover, a Cox proportional hazard regression model of univariate and multivariate analyses was performed using the constructed risk score model and known clinical information (age, stage, grade, and tumor residual disease).

### Nomogram Analysis and Robustness Verification

The Rms R package was used to perform the nomogram analysis by including those factors that may be significantly associated with the OS of patients with OC. A calibration plot was applied to estimate the discrimination between actual and nomogram-predicted OC probability. The sensitivity and specificity of the model were assessed by constructing a receiver operating characteristic (ROC) curve using the time ROC R package, and were used to analyze the predicted prognosis at 1, 3, and 5 years in patients with OC.

### Construction of a Network of miRNAs-m6A Regulators-m6A Target Genes

All miRNAs (sum of FPKM > 10) that were negatively correlated with the three selected m6A regulators (VIRMA, IGF2BP1, and HNRNPA2B1) were obtained from the GDC datasets (Pearson’s correlation coefficient < –0.1 and *P* < 0.05). Then, the miRNAs were further filtered through clinical significance based on their correlation with the 5-year survival rate of patients with OC. In addition, the related miRNAs possessing potential binding sites with the three selected m6A regulators were simultaneously predicted using the miRbase database with Target Score ≥ 80^3^ and the intersection with the results from the analysis in the previous step were collected. Furthermore, m6A-related genes involved in OC were obtained from the m6Avar database^4^ and further filtered based on Pearson’s correlation coefficients (*P* < 0.05) with the three selected m6A regulators. Forty-seven genes potentially coregulated by the three m6A regulators were further analyzed (Table 3) and annotated by Gene Ontology (GO) function and KEGG pathway enrichment analyses using Metascape^5^.

**TABLE 3:** Forty-seven genes were significantly correlated with three selected m6A RNA methylation regulators.

Gene name
HDAC1, USP24, JAK1, LRIG2, NRAS, SHE, SMG5, ZNF648, GALNT2, TTN, ZDBF2, TTLL4, NCL, GIGYF2, GTF2E1, SNX25, NR3C1, ZNF184, REV3L, SCRN1, BBS9, FLNC, BRWD3, ELF4, AIFM1, WRN, SPAG1, MPDZ, CENPP, RAG1, SYT7, MAP4K2, HSPA14, KIF20B, TAF5, SEC23IP, SYNM, ZNF594, KIF18B, ATRN, SLC2A10, CTCFL, DNMT1, MAN2B1, ZNF180, ITGB2, PCNT.

### Cell Lines and Culture

Human OC cells (SKOV3 and OVCAR3) and normal human ovarian surface epithelial cells (HOSEPiCs) were purchased from the Cell Bank of the Chinese Academy of Sciences (Shanghai, China). Cells were maintained at 37°C in a humidified atmosphere of 5% CO_2_ in RPMI-1640 medium supplemented with 10% fetal bovine serum (FBS) (Gibco, New York, NY, United States) and 100 U/mL penicillin/streptomycin (Corning, New York, NY, United States).

### Patient Samples

A total of 30 primary OC patients were enrolled in the study from April to June, 2021, which was approved by the Ethics Committee of The Third Affiliated Hospital of Zhengzhou University and written informed consent was obtained from all the patients. The patients were classified into the low(I + II) and high-stage (III + IV) groups on basis of the clinical information of TNM stage.

### RNA Extraction and qRT-PCR

Total RNA was isolated from OC cells and HOSEPiCs using TRIzol reagent (Invitrogen). RNA was reverse transcribed to cDNAs using the PrimeScript^TM^ RT reagent Kit with gDNA Eraser (Takara, Japan) and amplified by qRT-PCR with a SYBR Green Kit (Takara Bio Company) in an ABIPRISM 7500 Sequence Detection System (Applied Biosystems, Foster City, CA, United States), with the housekeeping gene GAPDH serving as an internal control. All primers were synthesized by Sangon Biotech (Shanghai, China), and the following primer sequences were used: IGF2BP1 forward primer: 5′-CAGGAGATGGTGCAGGTGTTTATCC-3′, reverse primer: 5′-GTTTGCCATA GATTCTTCCCTGAGC-3′; PTEN forward primer: 5′-TGGGCCCTGTACC ATCCCAAGT-3′, reverse primer: 5′-TGTGGCAACCACAGCCATCGT-3′; and GAPDH forward primer: 5′-TCTCTGCTCCTCCTGTTCGA-3′, reverse primer: 5′-GCGC CCAATACGACCAAATC-3′.

### Immunochemistry (IHC)

Paraffin-embedded tissue sections from OC specimens were given a heat pretreatment of 60°C for 1 h, then dewaxed in xylene, re-hydrated in an ethanol series (100–50%) and treated in 0.01 mol/L citrate buffer (pH 6.0) for antigen retrieval. The antibody used in the experiment included goat anti-VIRMA, IGF2BP1 and HNRNPA2B1 polyclonal antibody (Abcam, United States). The following experimental procedure was according to the manufacturer’s instructions. Besides, some expression data of the three selected m6A regulators (VIRMA, IGF2BP1, and HNRNPA2B1) in OC tissues on basis of the public database of The Human Protein Atlas^6^ were added.

### Plasmid Construction and Cell Transfection

SKOV3 and OVCAR3 cells were transiently transfected with the miR-196b-5p inhibitor or miRNA-NC (si-IGF2BP1 or si-NC) using Lipofectamine 3000 transfection reagent (Invitrogen, Carlsbad, CA, United States) according to the manufacturer’s instructions after being cultured in 6-well plates overnight. Forty-eight hours after transfection, the cells were harvested to detect the knockout efficiency via qPCR detection of miR-196b-5p (or IGF2BP1) expression. The miR-196b-5p inhibitor and miR-NC as well as si-IGF2BP1 and si-NC were designed and synthesized by GenePharma (Shanghai, China).

### Transwell Assay

For the migration assays, a 24-well Transwell chamber without Matrigel in the upper chamber was used. For the invasion assays, a same chamber where the upper chamber was coated with Matrigel (354230, BD, United States) was used. A total of 5.0 × 10^4^ transfected SKOV3 (or OVCAR3) cells in 200 μL of serum-free DMEM were seeded in the upper chamber, and 600 μL of medium containing 10% FBS was placed in the lower chamber. After an incubation for 24 h, cells on the upper membrane surface were removed with a cotton swab, and the invading cells that had traversed the membrane were stained with crystal violet and counted.

### Western Blot

For western blotting, the cells were fully lysed and total protein was extracted using RIPA lysis buffer (P0013, Beyotime, CA). Then, protein loading buffer was added to the protein sample and mixed, and the mixture was boiled for 10 min. Next, a 30 μg protein sample was added to each well, electrophoresed (at 80 V for 40 min and at 120 V for 80 min), and subsequently transferred to a PVDF membrane (at 200 mA for 60 min) (Immobilon-P Transfer Membrane, EMD Millipore Corporation, MA). The membrane was blocked with 5% skim milk for 2 h and then incubated with the primary antibody overnight at 4°C, followed by an incubation with the corresponding secondary antibody for 2 h at room temperature. Protein bands were visualized using an Odyssey scanner (LI-COR Biosciences, Lincoln, NE, United States). ImageJ software was used for the semiquantitative analysis. Primary antibodies against IGF2BP1, PTEN, and GAPDH were used.

### Dual Luciferase Reporter Assay

293T cells were seeded in 24-well plates at a density of 5 × 10^4^ cells/well and allowed to settle overnight. The next day, cells were cotransfected with pmirGLO-IGF2BP1-WT or-MUT reporter plasmids and the miR-196b-5p mimic. Twenty-four hours after transfection, 20 μL of the protein supernatant were added to 50 μL of the firefly luciferase substrate and mixed thoroughly. Then, the relative luciferase activity was measured using the Dual-Luciferase Reporter Assay System (Promega, Madison, WI, United States) and normalized to Renilla luciferase activity.

### RNA Stability Assays

SKOV3 (or OVCAR3) cells were transfected with si-IGF2BP1 or si-NC. Forty-eight hour after transfection, Actinomycin D (2 mg/mL, 129935, Millipore, United States) was added to the cell culture medium to inhibit mRNA biogenesis. The expression levels of PETN mRNA were detected at some fixed time points by RT-qPCR in the next 24 h. Then, the half-lives of PETN mRNA were evaluated and analyzed between the two groups.

### Statistical Analysis

The Wilcoxon rank test was applied to compare the expression levels of m6A regulators in ovarian tissues between the tumor group (GDC datasets) and normal group (GTEx datasets). Patients were separated into low-risk and high-risk groups by applying the median risk score (derived from the risk signature) as the cutoff value. Chi-square tests were applied to compare the distributions of patient age, survival status and grade between the two risk groups. One-way ANOVA or *t*-test was conducted to compare the risk scores of patients divided based on clinical or molecular-pathological features and to compare the risk scores of the signature for ovarian tissues with different clinicopathological characteristics. Univariate and multivariate Cox regression analyses were conducted to evaluate the prognostic value of the risk score and various clinical and molecular-pathological features. The prediction efficiency of the risk signature was tested by constructing a ROC curve. The Kaplan–Meier method with a two-sided log-rank test was applied to compare the OS of the patients in the low- and high-risk groups. All statistical analyses were executed utilizing R v3.4.1 and GraphPad Prism 8.

## Results

### Expression of 21 m6A RNA Methylation Regulators in OC Tissues

**FIGURE 2:**
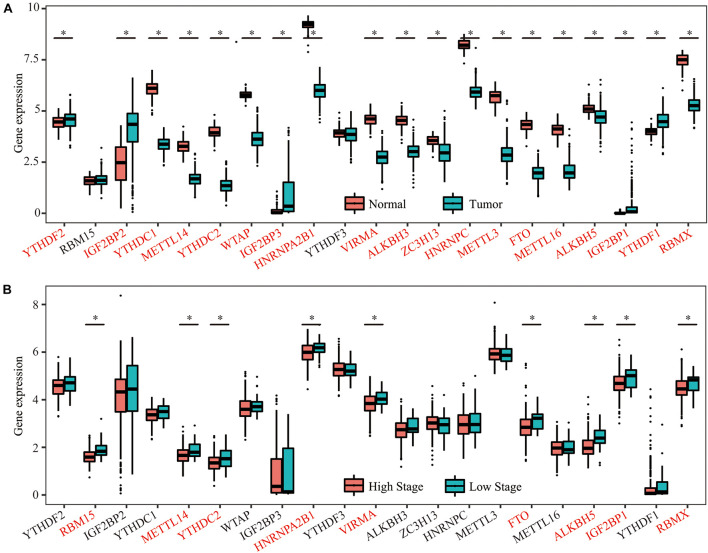
Expression of 21 m6A RNA methylation regulators in OC tissues and normal human ovarian tissues. **(A)** Five m6A regulators (YTHDF1, YTHDF2, IGF2BP1, IGF2BP2, and IGF2BP3) were upregulated, and 14 m6A regulators (METTL3, METTL14, METTL16, ZC3H13, WTAP, VIRMA, RBMX, YTHDC1, YTHDC2, HNRNPC, HNRNPA2B1, FTO, ALKBH3, and ALKBH5) were downregulated in the OC tissues compared with the control tissues. **(B)** Nine of the 21 m6A regulators (METTL14, YTHDC2, FTO, ALKBH5, HNRNPA2B1, VIRMA, IGF2BP1, RBM15, and RBMX) were significantly differentially expressed in the two groups, and all of them were downregulated in the high-stage group compared with the low-stage group *P < 0.05.

Considering the vital functions of m6A RNA methylation regulators in tumorigenesis and development, we first analyzed and compared the mRNA expression levels of the 21 key m6A RNA methylation regulators between OC and normal human ovarian tissues using datasets from the GDC and GTEx databases. Nineteen of the 21 m6A regulators were differentially expressed in OC tissues compared with the normal controls. Among these genes, 5 m6A regulators (YTHDF1, YTHDF2, IGF2BP1, IGF2BP2, and IGF2BP3) were upregulated, and 14 m6A regulators (METTL3, METTL14, METTL16, ZC3H13, WTAP, VIRMA, RBMX, YTHDC1, YTHDC2, HNRNPC, HNRNPA2B1, FTO, ALKBH3, and ALKBH5) were downregulated in the OC tissues compared with the control tissues (Figure 2A). We then divided the patients with OC into a low-stage group (stages I + II) and high-stage group (stages III + IV) according to their clinical characteristics and found that 9 of the 21 m6A regulators (METTL14, YTHDC2, FTO, ALKBH5, HNRNPA2B1, VIRMA, IGF2BP1, RBM15, and RBMX) were significantly differentially expressed in the two groups, and all of them were downregulated in the high-stage group compared with the low-stage group (Figure 2B). Therefore, the expression levels of 7 m6A regulators (METTL14, YTHDC2, FTO, ALKBH5, HNRNPA2B1, VIRMA, and RBMX) were lower in both the OC tissues and the high-stage group, indicating their potential function as tumor suppressors in OC tumorigenesis and development.

### Development of a Risk Signature Consisting of Three m6A Regulators Both in Train and Test Dataset

**FIGURE 3:**
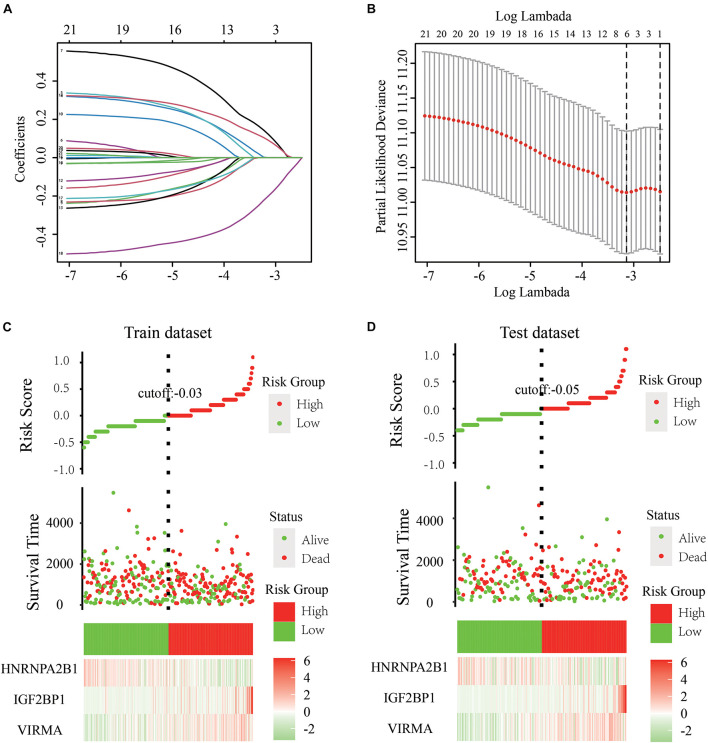
Development of a risk signature consisting of three m6A RNA methylation regulators to predict the prognosis of patients with OC. **(A,B)** LASSO Cox regression algorithm for the 21 regulators in the GDC dataset and obtained risk scores based on three m6A regulators (VIRMA, IGF2BP1, and HNRNPA2B1) as variables. **(C)** Using the median risk score (–0.03) as the cutoff point, all the 361 patients in the train dataset were divided into two groups, namely, the high-risk group and the low-risk group. **(D)** Using the median risk score (–0.05) as the cutoff point, 252 patients in the test dataset were also divided into two groups as previously described. A KM survival analysis was subsequently performed to evaluate the effectiveness of the three selected m6A regulators and to construct a risk model for the survival rate of patients with OC both in the train and test dataset.

We engaged the least absolute shrinkage and selection operator (LASSO) Cox regression algorithm to analyze the 21 regulators in the GDC dataset and better predict the clinical outcomes of patients with abnormal expression of m6A RNA methylation regulators; we obtained a risk score based on three m6A regulators (VIRMA, IGF2BP1, and HNRNPA2B1) as variables (Figure 3A,B). Using the median risk score (–0.03) as the cutoff point, we divided all patients (train dataset) into two groups, namely, the high-risk group and the low-risk group. We also constructed a test dataset consisting of 252 OC patients randomly selected from the GDC database. A KM survival analysis was subsequently performed to evaluate the effectiveness of the three selected m6A regulators and to construct a risk model for the survival rate of patients with OC both in the train and test datasets (Figures 3C,D).

**FIGURE 4:**
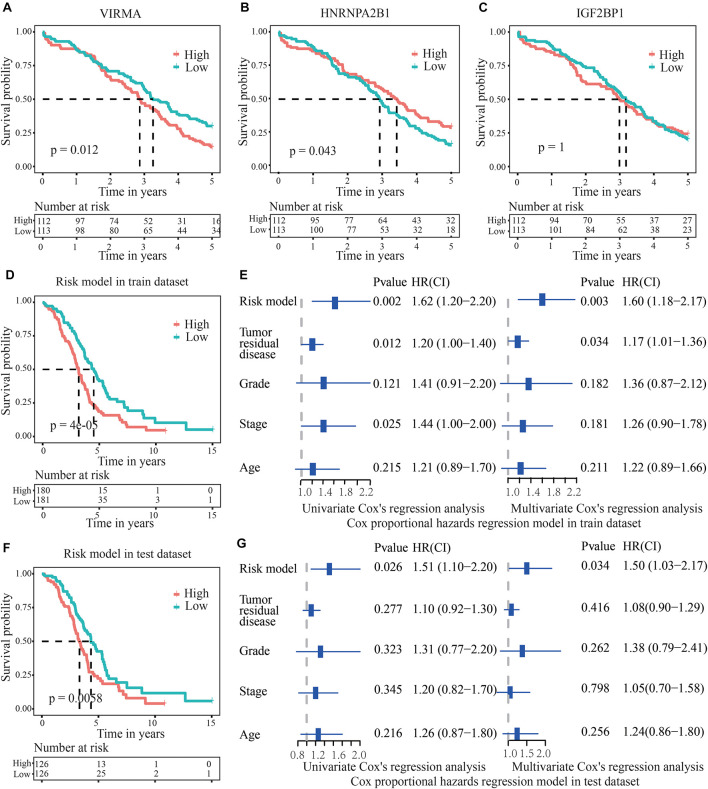
Validation of the accuracy of the risk score model to predict the prognosis of patients with OC. **(A,B)** The survival time of OC patients with 50% survival rate presenting low VIRMA expression or high HNRNPA2B1 expression was longer than that of the controls. **(C)** IGF2BP1 expression was not related to the 5-year survival rate of patients with OC. **(D)** In the train dataset, the survival time of OC patients with 50% survival rate in the low-risk group were obviously longer than those of patients in the high-risk group. **(E)** Univariate and multivariate analyses of patients with OC were executed to appraise whether clinicopathological characteristics were independent prognostic factors of patient outcomes. The univariate analysis indicated that the risk score model, stage and tumor residual disease were all independent factors predicting a poor prognosis for patients with OC. **(F)** In the test dataset, the survival time of OC patients with 50% survival rate in the low-risk group were also longer than those of patients in the high-risk group. **(G)** In the test dataset, the univariate analysis demonstrated that the risk model was also an independent factor predicting a poor prognosis for OC patients, which is consistent with the results from the train dataset.

The survival time of OC patients with 50% survival rate presenting low VIRMA expression or high HNRNPA2B1 expression was longer than that of the control patients (Figures 4A,B). However, the expression level of IGF2BP1 was not related to the 5-year survival rate of patients with OC (Figure 4C). However, when using the risk model based on the three m6A regulators in the train dataset, the survival time of OC patients with 50% survival rate in the low-risk group were obviously higher than those of patients in the high-risk group (*p* = 4e-05), suggesting its potential clinical significance (Figure 4D). Moreover, univariate and multivariate analyses of patients with OC were executed to appraise whether clinicopathological characteristics (including age, stage grade, tumor residual disease and risk model) were independent prognostic factors of patient outcomes. The univariate analysis applying the Cox proportional hazards model for all variables indicated that the risk model (*P* = 0.00174, 95% CI for the HR 1.2–2.2), stage (*P* = 0.025, 95% CI for the HR 1–2) and tumor residual disease (*P* = 0.0124, 95% CI for the HR 1–1.4) were all independent factors predicting a poor prognosis for patients with OC (Figure 4E). As for the test dataset, the survival time of OC patients with 50% survival rate in the low-risk group were also obviously higher than those of patients in the high-risk group (*p* = 0.0058, Figure 4F). In addition, univariate analyses of patients with OC applying the Cox proportional hazards model for all variables also demonstrated that the risk model (*P* = 0.026, 95% CI for the HR 1.1–2.2) was also an independent factor predicting a poor prognosis for patients with OC (Figure 4G), which is consistent with the results from the train dataset.

### Establishment of a Nomogram Based on Clinicopathological Characteristics, Including the Risk Model

**FIGURE 5:**
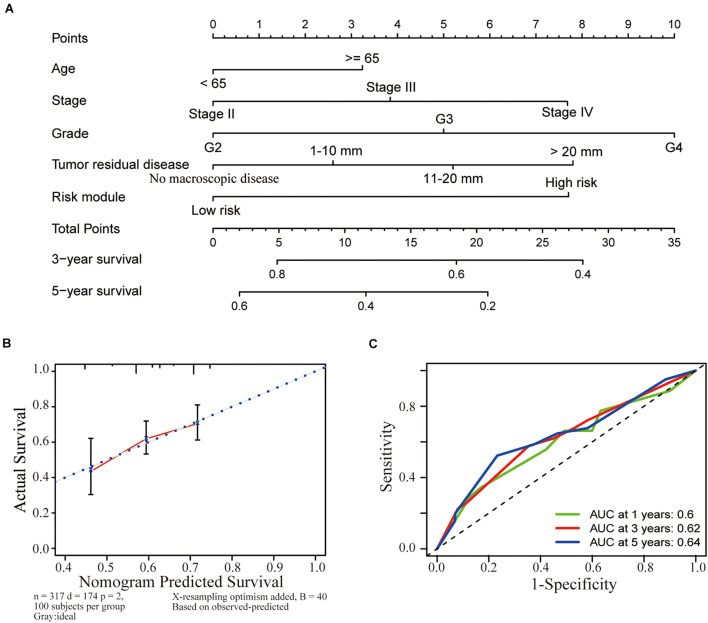
Establishment of a nomogram based on clinicopathological characteristics, including the risk model. **(A)** A nomogram was established to identify the relationship between the survival time and clinicopathological characteristics (including age, stage, grade, residual tumor disease, and risk model). The survival probability of patients with OC at 3 and 5 years was estimated by calculating the total points corresponding to the nomogram. **(B)** The nomogram had favorable predictive power for the 3-year and 5-year survival of patients with OC. **(C)** A ROC curve analysis was conducted to determine the accuracy of the prediction obtained from the risk score model. The calculation of the area under the curve (AUC) was 0.6 for 1 year, 0.62 for 3 years, and 0.64 for 5 years.

A nomogram was established to help us to understand the relationship between the survival time and clinicopathological characteristics (including age, stage, grade, residual tumor disease, and risk model). By calculating the total points corresponding to the nomogram, we were able to estimate the survival probability of patients with OC at 3 and 5 years (Figure 5A). Meanwhile, calibration plots were applied to verify the predictive power of the nomogram. The nomogram displayed favorable predictive power for the 3-year and 5-year survival of patients with OC (Figure 5B). Furthermore, a time-dependent ROC curve analysis was used to detect the accuracy of the prediction generated by the risk score model. The calculation of the area under the curve (AUC) was 0.6 for 1 year, 0.62 for 3 years, and 0.64 for 5 years (Figure 5C), suggesting that the prognostic risk score had moderate accuracy.

### Predicted miRNAs Targeted the Three Selected m6A RNA Methylation Regulators

**FIGURE 6:**
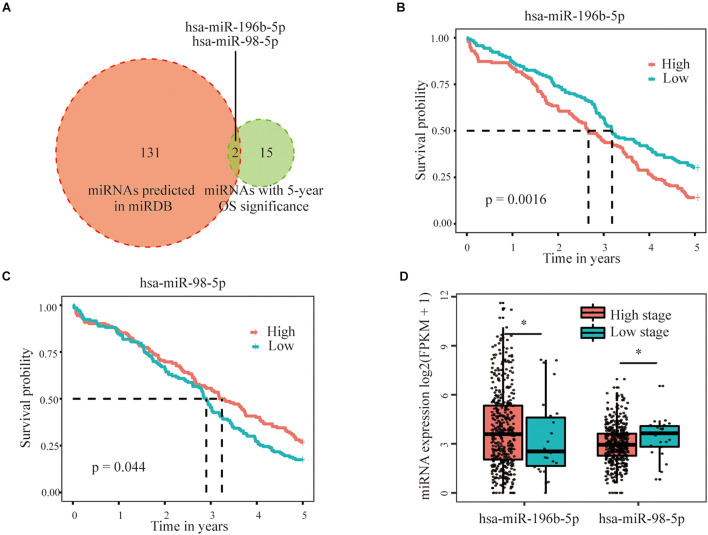
Predicted miRNAs targeted the three selected m6A RNA methylation regulators. **(A)** Two miRNAs, namely, hsa-miR-196b-5p and hsa-miR-98-5p, with potential binding sites in the IGF2BP1 sequence that were significantly related to the prognosis of patients with OC were selected based on miRbase and survival relationships. **(B)** Patients with OC presenting high hsa-miR-196b-5p expression showed an obviously decreased survival rate compared with those with low hsa-miR-196b-5p expression, indicating its oncogenic role in OC. **(C)** Patients with OC presenting high hsa-miR-98-5p expression showed an increased survival rate compared with those with low hsa-miR-98-5p expression, suggesting its potential tumor-suppressive function in OC. **(D)** The expression of hsa-miR-196b-5p was increased, but hsa-miR-98-5p expression was decreased in the high-stage group compared with the low-stage group *P < 0.05.

Three hundred fifty-eight miRNAs that were negatively correlated with the expression of three selected m6A regulators were obtained from the GDC datasets (Pearson’s correlation coefficient < –0.1 and *p* < 0.05) to further identify the upstream regulators of the three important m6A regulators (VIRMA, IGF2BP1, and HNRNPA2B1). Notably, 17 miRNAs were further filtered out based on their correlation with the 5-year survival rate of patients with OC. In addition, 40 miRNAs possessing potential binding sites for HNRNPA2B1, 46 miRNAs with binding sites for VIRMA, and 68 miRNAs with binding sites for IGF2BP1 (a total of 133 non-repetitive miRNAs) were predicted based on the bioinformatic analysis of the miRbase database. When taking the intersection of the results from the analysis in the previous step, only two miRNAs remained, namely, hsa-miR-196b-5p and hsa-miR-98-5p, which had potential binding sites in IGF2BP1 and were significantly related to the prognosis of patients with OC (Figure 6A). Through further expression and survival correlation analyses, we found that the expression of hsa-miR-196b-5p was increased in the high-stage group compared with that in the low-stage group, and patients with OC presenting high hsa-miR-196b-5p expression experienced an obviously decreased survival rate compared with those with low hsa-miR-196b-5p expression (*p* = 0.0016), indicating its oncogenic function in OC (Figures 6B,C). Meanwhile, hsa-miR-98-5p expression was decreased in the high-stage group compared with that in the low-stage group, and patients with OC presenting high hsa-miR-98-5p expression experienced an increased survival rate compared with those with low hsa-miR-98-5p expression (*p* = 0.044), suggesting its potential tumor-suppressive function in OC (Figures 6B,D).

### Construction of a Network of miRNAs-m6A Regulators-m6A Target Genes

**FIGURE 7:**
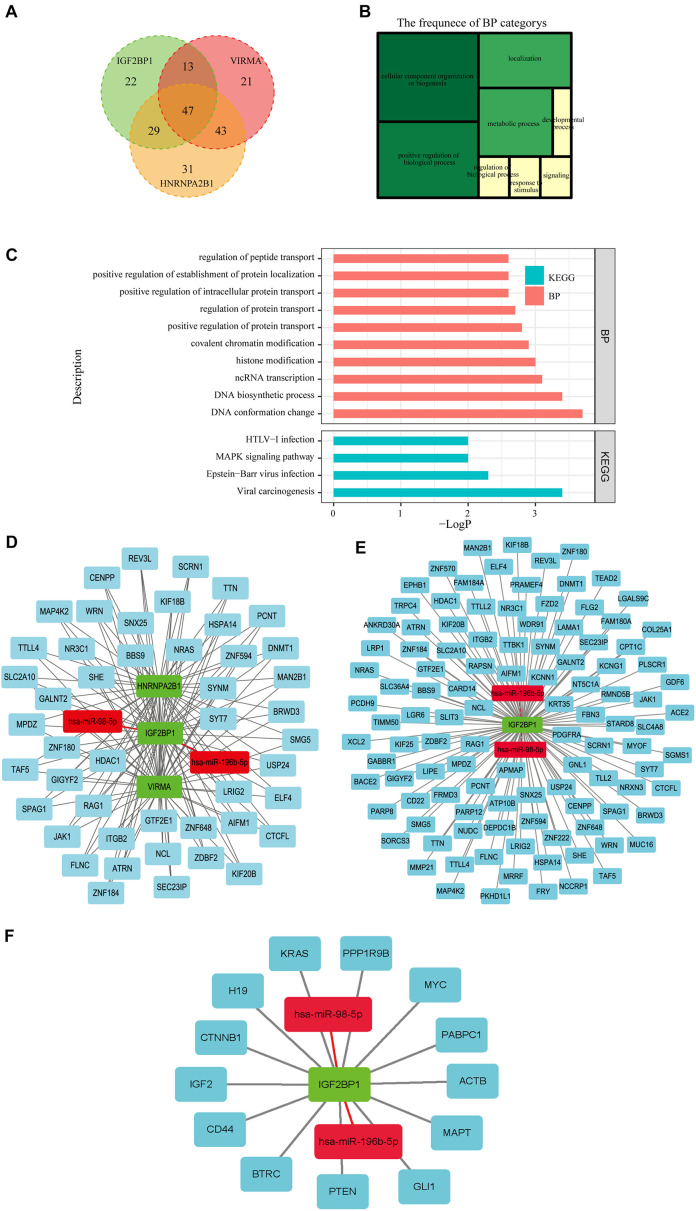
Construction of a network of miRNAs-m6A regulators-m6A target genes. **(A)** Forty-seven m6A target genes potentially coregulated by the three m6A regulators (VIRMA, IGF2BP1, and HNRNPA2B1) were obtained from m6Avar, an updated database of functional variants involved in RNA modifications. **(B,C)** The GO analysis showed that the biological processes of the 47 genes were mainly enriched in cellular components organization and biogenesis, and positively regulated biological processes such as protein transport and localization. The KEGG pathway analysis indicated that these genes were mainly enriched in viral carcinogenesis and the MAPK signaling pathway. **(D)** A regulatory network of miRNAs-m6A regulators-m6A target genes was constructed, which was composed of two miRNAs, three m6A regulators, and forty-seven mRNAs. **(E)** A regulatory network composed of two miRNAs, IGF2BP1, and110 m6A target genes was constructed. **(F)** A regulatory network of two miRNAs, IGF2BP1, and 10 target genes that has been reported previously.

Three hundred eighty m6A RNA methylation-related genes involved in OC were further obtained from m6Avar, an updated database of functional variants involved in RNA modifications, and further filtered based on Pearson’s correlation coefficients (*p* < 0.05) with the three selected m6A regulators (VIRMA, IGF2BP1, and HNRNPA2B1) to construct the regulatory network among miRNAs, m6A regulators, and m6A target genes. One hundred twenty-three genes related to VIRMA, 110 genes related to IGF2BP1 and 148 genes related to HNRNPA2B1 were obtained, and 47 of these genes that are potentially coregulated by the three m6A regulators were further analyzed by performing GO function and KEGG pathway enrichment analyses (Figure 7A). The GO analysis showed that the biological processes of the 48 genes were mainly enriched in cellular components organization and biogenesis, and positively regulated biological processes such as protein transport and localization (Figures 7B,C). The KEGG pathway analysis indicated that these genes were mainly enriched in viral carcinogenesis and the MAPK signaling pathway, which may indicate their relationship with OC development and progression (Figure 7C). Then, a regulatory network of miRNAs-m6A regulators-m6A target genes was constructed, which was composed of two miRNAs, three m6A regulators, and forty-seven mRNAs (Figure 7D). In addition, networks of two selected miRNAs (miR-196b-5p and miR-98-5p), IGF2BP1, and 110 predicted m6A target genes or 10 previously reported m6A target genes were constructed (Figures 7E,F).

### One of the miRNA-m6A Regulator-m6A Target Gene Pathways Was Chosen for Preliminary Verification

**FIGURE 8:**
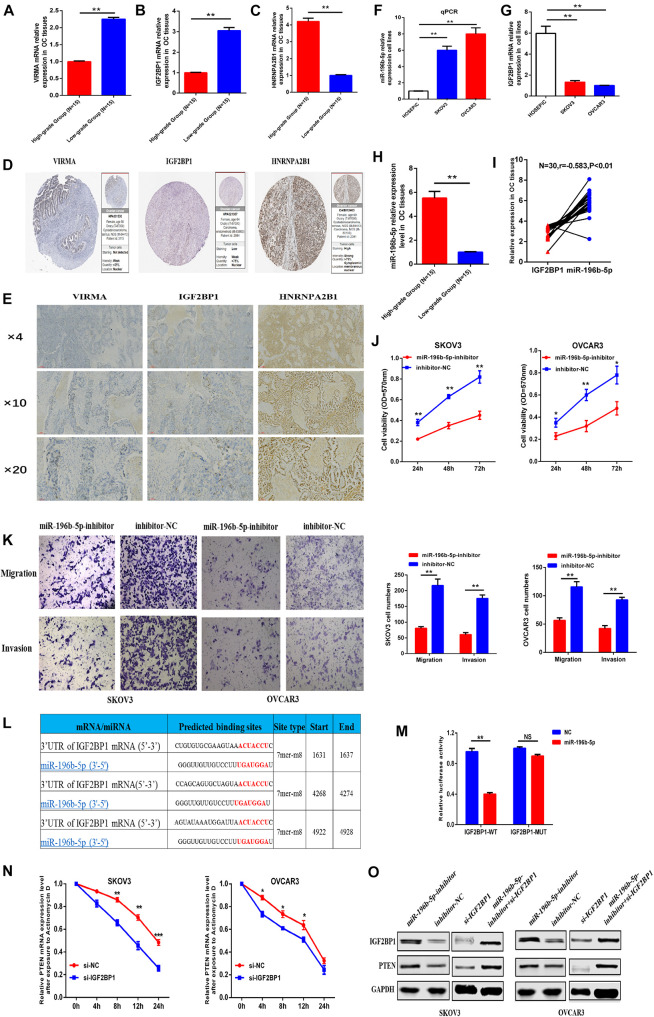
One of the miRNA-m6A regulator-m6A targeted gene pathways was chosen for preliminary verification. **(A–C)** mRNA expression of VIRMA, IGF2BP1, and HNRNPA2B1 in the high-stage and low-stage OC tissues by PCR. **(D)** Protein expression of VIRMA, IGF2BP1, and HNRNPA2B1 in OC tissues by IHC. **(E)** Protein expression of VIRMA, IGF2BP1, and HNRNPA2B1 in OC tissues from public database of The Human Protein Atlas. **(F,G)** miR-196b-5p was upregulated, and IGF2BP1 was downregulated in ovarian cancer cells compared with control cells. **(H,I)** miR-196b-5p, negatively correlated with IGF2BP1(*r* = –0.583, *P* < 0.05), was upregulated in the OC tissues of high-stage group than the controlled. **(J)** The CCK-8 assay showed that the proliferation of SKOV3 or OVCAR3 cells with decreased miR-196b-5p expression induced by miRNA inhibitor transfection was obviously decreased compared with that of the control group. **(K)** Transwell assays revealed obvious decreases in the migration and invasion of SKOV3 or OVCAR3 cells with miR-196b-5p knockdown induced by miRNA inhibitor transfection compared with the control group. **(L,M)** Dual luciferase reporter gene experiments confirmed the predicted binding sites between miR-196b-5p and IGF2BP1. **(N)** After exposure to Actinomycin D to inhibit the novel mRNA synthesis, the degradation rate of PTEN mRNA was significantly faster in the SKOV3 or OVCAR3 cells transfected with si-IGF2BP1 than that in the cells transfected with si-NC. **(O)** Downregulation of miR-196b-5p increased the levels of the IGF2BP1 and PTEN proteins. And the protein level of IGF2BP1 and PTEN downregulated by si-IGF2BP1 transfection in the SKOV3 or OVCAR3 cells could be partly relieved by transfection of miR-196b-5p-inhibitor *P < 0.05, **P < 0.01, and ***P < 0.001.

To identify the reliability of the results based on bioinformatics analysis, we first examined the expression of the three selected m6A regulators (VIRMA, IGF2BP1, and HNRNPA2B1) in OC tissues. PCR and IHC assays showed that the expression of VIRMA and IGF2BP1 were down-regulated in the high-stage OC tissues compared with that of the low-stage OC tissues, but HNRNPA2B1 expression was up-regulated in the high-stage OC tissues compared with that of the low-stage OC tissues (Figures 8A–D). Besides, we found that some IHC data of the three selected m6A regulators (VIRMA, IGF2BP1, and HNRNPA2B1) in OC tissues on basis of the public database of The Human Protein Atlas was consistent with our results (Figure 8E).

To further validate the credibility of our constructed network, one of the miRNA-m6A regulator-m6A related gene pathways, namely, miR-196b-5p-IGF2BP1-PTEN, was chosen for preliminary verification to further assess the credibility and reliability of our constructed regulatory network. As the function of miR-196b-5p and its relationship with IGF2BP1 in OC are still unclear, we first examined the expression levels of miR-196b-5p and the IGF2BP1 mRNA in OC cells and normal ovarian epithelial cells and found that miR-196b-5p was upregulated and IGF2BP1 was downregulated in ovarian cancer cells compared with control cells (Figures 8F,G). We also examined the miR-196b-5p expression in the 30 OC tissues previously described and found that miR-196b-5p, negatively correlated with IGF2BP1(*r* = –0.583, *P* < 0.05), was upregulated in the OC tissues of high-stage group than the controlled (Figures 8H,I). Further CCK-8 assays were then conducted to identify the function of miR-196b-5p in OC cell proliferation, and the proliferation rate of SKOV3 or OVCAR3 cells with decreased miR-196b-5p expression following the transfection of an miRNA inhibitor was obviously decreased compared with that of the control group (Figure 8J). Similar results of migration and invasion were observed in the Transwell assays using SKOV3 or OVCAR3 cells with decreased miR-196b-5p expression (Figure 8K). Dual luciferase reporter gene experiments also confirmed the predicted binding sites between miR-196b-5p and IGF2BP1 (Figures 8L,M). Considering the vital role of m6A regulators in RNA stability and metabolism, the mRNA stability assay was preformed to further the regulatory relationship between IGF2BP1 and PTEN and the results showed that after exposure to Actinomycin D to inhibit the novel mRNA synthesis, the degradation rate of PTEN mRNA was significantly faster in the SKOV3 or OVCAR3 cells transfected with si-IGF2BP1 than that in the cells transfected with si-NC (Figure 8N). In addition, downregulation of miR-196b-5p increased the levels of the IGF2BP1 and PTEN proteins. And the protein level of IGF2BP1 and PTEN was downregulated in the SKOV3 or OVCAR3 cells transfected with si-IGF2BP1, which could be partly relieved by transfection of miR-196b-5p-inhibitor (Figure 8O). On basis of the above results, we initially established the regulatory pathway of miR-196b-5p-IGF2BP1- PTEN in OC, suggesting the reliability of our constructed regulatory network to some extent.

## Discussion

m6A RNA methylation achieved by different regulators is one of the most important RNA modifications and could affect gene expression in terms of the transcriptome layer to further participate in the regulation of various physiological and pathological cellular processes (Karthiya and Khandelia, 2020). Based on accumulating evidence, dysregulation of m6A RNA methylation regulators is closely related to the occurrence and development of many diseases, such as cardiovascular diseases (Wu et al., 2020), neurodegenerative diseases (Du et al., 2019), immune-related diseases, and cancers (Maity and Das, 2016). Considering the universality and importance of m6A RNA methylation, it is likely to provide novel insights and clues to identify the pathogenesis of many cancers, including OC (He, 2021). Several reports have assessed the role of m6A RNA methylation regulators, such as METTL3 (Bi et al., 2021), FTO (Huang et al., 2020b; Liu et al., 2020) and YTHDF1, in OC progression. In recent years, an increasing number of novel m6A RNA methylation regulators have been identified. However, previous studies have mainly focused on the function and mechanism of a few classic m6A regulators, such as METTL3. The expression and functional role of key m6A RNA methylation regulators in the tumorigenesis and progression of OC and their related regulatory networks have not yet been comprehensively analyzed.

In the current study, we analyzed and compared the expression of 21 key m6A RNA methylation regulators in OC and normal tissues and observed lower expression levels of 7 m6A regulators (METTL14, YTHDC2, FTO, ALKBH5, HNRNPA2B1, VIRMA, and RBMX) both in OC tissues and in the high-stage group, indicating their potential functions as tumor suppressors in OC tumorigenesis and development. Next, we explored the prognostic value of each m6A RNA methylation regulator and developed a risk score model applying three chosen m6A RNA methylation regulators, VIRMA, IGF2BP1, and HNRNPA2B1, which were selected by the LASSO Cox regression analysis. Based on this signature, we divided all patients into a high-risk group and a low-risk group, and a subsequent KM survival analysis showed that the 5-year and 10-year survival rates of patients with OC in the low-risk group were obviously higher than those of patients in the high-risk group, which may suggest its potential clinical significance. Then, we established a nomogram that assimilated some clinical parameters, including the risk model associated with OC prognosis, and performed univariate and multivariate analyses to assess the prognostic value of our constructed risk score model. Moreover, the ROC analysis further confirmed the moderate accuracy of the prediction obtained using our risk score model. In addition, we constructed a regulatory network of miRNAs-m6A regulators-m6A target genes, including two miRNAs, three m6A regulators, and forty-seven mRNAs, based on a systematic bioinformatic analysis. Finally, we chose and initially validated one of the pathways, namely, miR-196b-5p-IGF2BP1-PTEN, in OC, which may represent a potential biomarker or therapeutic target in OC in the future.

Several reports have established a prognostic model of cancer based on the gene signature derived from m6A regulators. For example, Zhao et al. (2020) identified and constructed a three-m6A-related gene (METTL3, METTL14, and HNRNPA2B1)-based risk score model as a potential prognostic biomarker for clear cell renal cell carcinoma. Huang et al. (2020a) developed a prognostic model consisting of five m6A-associated genes (KIAA1429, METTL3, YTHDF1, YTHDF2, and ZC3H13) for hepatocellular carcinoma and confirmed the good performance of this model. In addition, Wang Z. et al. (2020) also constructed a five-gene signature (METT14, WTAP, HNRNPC, YTHDF1, and IGF2BP2) derived from m6A regulators to improve the prediction of the prognosis of patients with neuroblastoma (Wang Z. et al, 2020). In this study, we developed a risk score model incorporating three chosen m6A RNA methylation regulators, VIRMA, IGF2BP1, and HNRNPA2B1, selected by LASSO Cox regression analysis and further confirmed its accuracy by performing nomogram, Cox regression and ROC curve analyses.

Among these regulators, vir-like m6A methyltransferase associated (VIRMA), a “writer” of m6A regulators, promotes the progression of cancer and is associated with shorter survival for patients with multiple types of cancer, such as kidney renal clear cell carcinoma, kidney renal papillary cell carcinoma, and papillary thyroid carcinoma (Zhu et al., 2021). However, no reports have examined the correlation between VIRMA expression and OC progression. Here, VIRMA was expressed at lower levels both in OC tissues and in the high-stage group, indicating its potential function as a tumor suppressor in OC tumorigenesis, which was confirmed in our collected OC tissue samples by PCR and IHC. Heterogeneous nuclear ribonucleoprotein A2/B1 (HNRNPA2B1) is an important “reader” of m6A regulators that belongs to the A/B subfamily of ubiquitously expressed heterogeneous nuclear ribonucleoproteins (hnRNPs) and is associated with premRNAs in the nucleus and appears to influence premRNA processing and other aspects of mRNA metabolism and transport (Alarcón et al., 2015). Some previous studies have reported the role of HNRNPA2B1 as an oncogene in OC progression. For example, Yang et al. (2020) found that the loss of hnRNPA2B1 inhibits malignancy and promotes apoptosis in OC by downregulating Lin28B expression. Wang J. M. et al. (2020) showed that ISG15 suppresses the translation of ABCC2 via the ISGylation of hnRNPA2B1 and increases drug sensitivity in cisplatin-resistant OC cells. In the current study, we also found that hnRNPA2B1 expression was up-regulated in the OC tissues of high-stage group than the controlled by the PCR and IHC validation, which may indicate its potential role as an oncogene in the OC progression.

Regarding another “reader” of m6A regulators, insulin-like growth factor 2 mRNA binding protein 1 (IGF2BP1), some reports have previously documented its vital role in OC progression. For example, Gu et al. (2004) showed that increased IGF2BP1 expression is associated with an advanced clinical stage and poor prognosis in patients with OC. As shown in the study by Bley et al. (2021), IGF2BP1 is a targetable SRC/MAPK-dependent driver of invasive growth in OC. In addition, Qin et al. (2017) reported that the restoration of miR-708 sensitizes OC cells to cisplatin via the IGF2BP1/Akt pathway. In the current study, IGF2BP1 expression was downregulated in OC tissues and cells. We also predicted the upstream miRNAs of IGF2BP1 using the miRbase database and found that only hsa-miR-196b-5p and hsa-miR-98-5p targeted IGF2BP1 and were significantly related to the prognosis of patients with OC. Of these miRNAs, miR-98-5p has been reported to be related to cisplatin resistance and a poor prognosis of patients with OC (Wang et al., 2018; Guo et al., 2019; Dong et al., 2020), but no studies have examined miR-196b-5p in OC. Then, we further examined the expression of miR-196b-5p in OC cells and tissues, and found that miR-196b-5p, negatively correlated with IGF2BP1, was upregulated in OC cells and tissues compared with control. Transwell assays also revealed that miR-196b-5p increased the migration and invasion of OC cells by upregulating the expression levels of the IGF2BP1 and PTEN proteins. Subsequent dual luciferase reporter gene experiments also confirmed the predicted binding sites between miR-196b-5p and IGF2BP1. After exposure to Actinomycin D to inhibit the novel mRNA synthesis, the degradation rate of PTEN mRNA was significantly faster in the si-IGF2BP1 group than that in the si-NC group, which demonstrated the role of IGF2BP1 in maintaining the stability of PETN mRNA. Besides, the protein level of IGF2BP1 and PTEN downregulated by si-IGF2BP1 transfection in the SKOV3 or OVCAR3 cells could be partly rescued by transfection of miR-196b-5p-inhibitor, which suggest the mutually regulatory relationship among them. We initially established the regulatory pathway of miR-196b-5p-IGF2BP1-PTEN in OC, suggesting the reliability of our constructed regulatory network to some extent.

However, there are still some limitations in the current study. Firstly, we failed to verify our constructed risk model with external data from GEO or other databases because the data from GEO based on the previously lower version of microarray lack the expression data of all the 21 m6A genes to server as the test data. Secondly, we just examined the expression of the m6A regulators in the current study and some functional experiments will be added to identify the direct molecular function of them in the subsequent research.

## Conclusion

In conclusion, we established a three-m6A-related gene (VIRMA, IGF2BP1, and HNRNPA2B1)-based risk score model as a potential prognostic biomarker for OC. ROC curve, nomogram, and univariate and multivariate Cox regression analyses were also performed to confirm the moderate accuracy of our risk score model, indicating that the model may be reliably employed to predict the prognosis of patients with OC. In addition, a regulatory network of miRNAs-m6A regulators-m6A target genes was constructed based on a systematic bioinformatic analysis, and one of the pathways, miR-196b-5p-IGF2BP1-PTEN, was initially validated in OC, suggesting its utility as a potential biomarker or therapeutic target in OC in the future.

## Data Availability Statement

The datasets presented in this study can be found in online repositories. The names of the repository/repositories and accession number(s) can be found in the article/supplementary material.

## Ethics Statement

The studies involving human participants were reviewed and approved by Ethics Committee of The Third Affiliated Hospital of Zhengzhou University. The patients/participants provided their written informed consent to participate in this study.

## Author Contributions

QL and Y-NC carried out the data download from a public database and bioinformatics analysis. QL and LY collected the OC samples and carried out the IHC assays. WB-J and ZF carried out the cell biology experiment. Y-HZ and F-YL carried out the molecular biology experiment. JY, Z-AZ, and C-CR participated in the design of the study and performed the statistical analysis. QL drafted the manuscript. C-CR helped to correct the manuscript. All authors contributed to the article and approved the submitted version.

## Conflict of Interest

The authors declare that the research was conducted in the absence of any commercial or financial relationships that could be construed as a potential conflict of interest.

## Publisher’s Note

All claims expressed in this article are solely those of the authors and do not necessarily represent those of their affiliated organizations, or those of the publisher, the editors and the reviewers. Any product that may be evaluated in this article, or claim that may be made by its manufacturer, is not guaranteed or endorsed by the publisher.

## Footnotes

1
https://portal.gdc.cancer.gov/

2
https://www.gtexportal.org/home/datasets

3
http://www.mirbase.org/

4
http://m6avar.renlab.org/

5
https://metascape.org/gp/index.html#/main/step1

6
https://www.proteinatlas.org/
